# Imbalance of Th17/Tregs in rats with smoke inhalation-induced acute lung injury

**DOI:** 10.1038/srep21348

**Published:** 2016-02-17

**Authors:** Fan Zhang, Mian-yang Li, Ya-ting Lan, Cheng-bin Wang

**Affiliations:** 1 Department of Clinical Laboratory Medicine, Chinese People’s Liberation Army General Hospital & Postgraduate Medical School, Beijing 100853, People’s Republic of China

## Abstract

T helper (Th) 17 cells and CD4^+^ CD25^+^ regulatory T (Treg) cells are supposed to be critically involved in regulating autoimmune and inflammatory diseases. The aim of this study was to investigate the Th17/Treg pattern in rats with gunpowder smog-induced acute lung injury. Wistar rats were equally randomized to three groups: normal control group, ALI 6 h group (smoke inhalation for 6 h) and ALI 24 h group (smoke inhalation for 24 h). We observed changes in cell counting in bronchoalveolar lavage fluid (BALF), alveolar-capillary membrane permeability and lung tissue pathology. Moreover, rats in ALI 6 h and ALI 24 h group showed increased expression of Th17 cell and related cytokines (IL-17 A, IL-6, TGF-β and IL-23). Meanwhile, Treg prevalence and related cytokines (IL-10, IL-2 and IL-35) were decreased. Consequently, the ratio of Th17/Treg was higher after smoke inhalation. Additionally, Th1 cell decreased while Th2 cell increased at 6 h and 24 h after smoke inhalation. In conclusion, Th17/Treg imbalance exists in rats with smoke inhalation-induced acute lung injury, suggesting its potential role in the pathogenesis of this disease.

Smoke inhalation injury is generally defined as inhalation of thermal or chemical irritants^1^, with a high incidence of pulmonary complications and mortality in burn patients and soldiers^2^,^3^. Military operations are complex, dynamic, and always dangerous because of toxic industrial chemicals and materials such as gunpowder. In military activities, gunpowder is used to shield the soldiers, attack enemy or simulate battlefield environments. Gunpowder can generate large amounts of toxic gases and particles during explosion and combustion. Smoke inhalation is the leading cause of acute lung injury (ALI), acute respiratory distress syndrome (ARDS), or even serious respiratory failure in military personnel. Despite decades of intense research, the molecular mechanisms involved in the pathogenesis of smoke inhalation-induced acute lung injury are poorly defined. It is generally believed that inflammatory cells and release of inflammatory mediators, especially neutrophils and macrophages, are mandatory in the pathological process of smoke inhalation-induced acute lung injury^4^,^5^. However, the role of adaptive immune cells in this disease is less well defined. A recent study indicated that lymphocyte-deficient mice were unable to increase neutrophils in response to lipopolysaccharide, suggesting that T lymphocytes may contribute to pulmonary inflammatory pathways in ALI^6^. Most recently, CD4^+^ T lymphocytes, especially regulatory T (Treg) cells and T helper (Th) 17 cells, have become an active topic of research in the pathogenesis or resolution of ALI/ARDS^7^,^8^,^9^,^10^,^11^. Both cell types are generated from naive T cells that require transforming growth factor (TGF)-β with opposing actions^12^.

CD4^+^ CD25^+^ Foxp3^+^ Tregs plays an anti-inflammatory role mainly by contact-dependent suppression or releasing inhibitory cytokines such as IL-10 and TGF-β on other immune cells, including CD4^+^ and CD8^+^ T cells, B cells, natural killer (NK) cells and dendritic cells^13^. Reduced generation or deficient function of Tregs has been reported in a number of autoimmune diseases^14^. Notably, D’Alessio and colleagues’ study showed that Treg modifies innate immune responses during resolution of lung injury, suggesting its potential role in treating ALI^15^. Particularly, Treg contributes to the resolution of fibroproliferation in ALI^8^. However, a recent study showed that an increased ratio of Tregs is an independent risk factor for 30-day mortality in bronchoalveolar lavage fluid (BALF) of patients with ARDS on admission^7^.

In contrast to Tregs, Th17 cell plays a potent proinflammatory role by producing the signature cytokine IL-17A. Th17 cells have been reported to be implicated in autoimmune and lung diseases in animal and clinical studies^16^,^17^,^18^,^19^,^20^. Notably, Th17 cells and IL-17 increased in patients with ARDS compared to control group^21^. Additionally, studies revealed that IL-17A could act as a pro-inflammatory cytokine and may play an important role in ALI induced by lipopolysaccharide or H1N1 influenza virus^22^,^23^. Moreover, *Bordetella pertussis* could promote lung injury by increasing Th17 immunity^24^. Therefore, CD4^+^ T-lymphocyte-based therapeutic strategies may be more meaningful in ALI and provide us with a much broader intervention window. Indeed, studies have demonstrated that both losartan and alanylglutamine may protect mice from lipopolysaccharide-induced lung injury by suppressing Th17 immune responses and modulating the Th17/Treg balance in favor of Tregs, respectively^11^,^25^. It has been shown that the balance between Th17 and Treg is vital in the development of autoimmune and inflammatory diseases. However, the role of Th17/Treg balance in smoke inhalation-induced acute lung injury is currently unknown. Therefore, the aim of this study was to investigate the Th17/Treg pattern in rats with gunpowder smog-induced acute lung injury.

## Results

### Effects of different dosage of gunpowder and exposure time on survival

Mortality of rats increased with the addition of gunpowder dosage. When rats were exposed to smoke for 21 min, the mortality increased with dosage of gunpowder. There were no rats dead when exposure to smoke generated by 5 g of gunpowder. When the dosage of gunpowder increased from 7.5 to 10 g, the mortality increased from 12.5% to 77.8% within 4 days. The mortality was 100% when exposure to smoke generated by 15 g of gunpowder.

Median lethal dose of gunpowder ranges from 8 to 10 g. Therefore, we chose 10 g of gunpowder to generate smoke. When rats were exposed to smoke generated by 10 g of gunpowder, the mortality increased with prolongation of exposure time. There were no rats dead within 4 days when exposure for 8 min. In addition, 21 min of exposure resulted in 77.8% mortality within 4 days. Therefore, exposure to the smoke generated by 10 g gunpowder for 8 min was the optimum injury condition.

### Smoke composition

The concentration of O_2_, CO, H_2_S was measured (Table 1). The temperature in the smoke chamber was also shown in Table 1.

**Figure 1:**
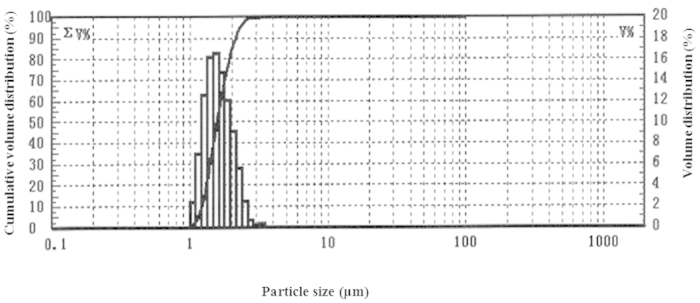
Particulate matters distribution of the gunpowder smog.

Particulate matter (PM) is important component of the smoke from incomplete combustion. In this study, we measured the PM distribution in the gunpowder smoke. As shown in Fig. 1, the results showed that the main components were PM2.5 (particles with an aerodynamic diameter smaller than 2.5 μm).

### Lung vascular permeability

**Figure 2:**
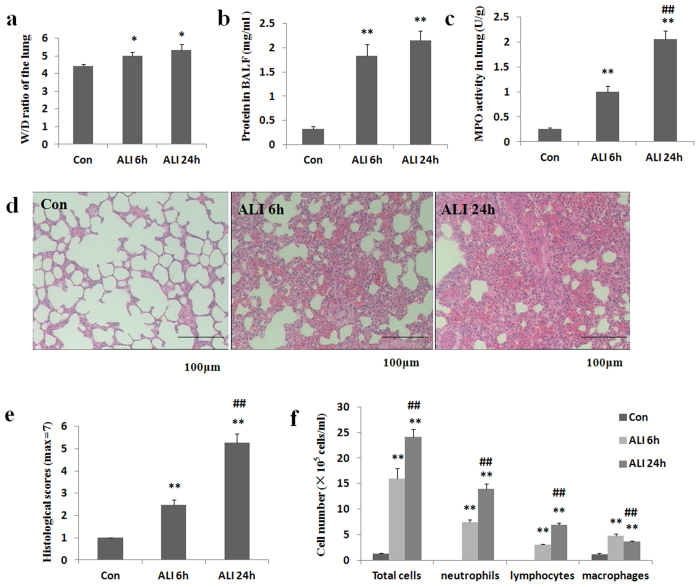
Gunpowder smog induces pulmonary inflammation. Lung vascular permeability parameters including lung W/D weight ratio (**a**) and protein concentration in BALF (**b**) were determined after smoke inhalation. (**c**) Myeloperoxidase (MPO) activity in lung homogenates was determined after smoke inhalation. (**d**) Representative images of hematoxylin and eosin–stained lung sections showed obvious thickening of the alveolar wall, interstitial edema and infiltration of inflammatory cells in rats with smoke inhalation. Scale bars, 100 μm. (**e**) Histological scores of lung histopathology. (**f**) Gunpowder smog induces inflammatory cell infiltration in BALF. The number of total inflammatory cells, neutrophils, lymphocytes and macrophages in BALF was determined after smoke inhalation. Con (normal control group, ambient air inhalation, n = 10), ALI 6 h (ALI group, smoke inhalation for 6 h, n = 10), ALI 24 h (ALI group, smoke inhalation for 24 h, n = 10); data are presented as mean ± S.E.M. *P < 0.05 and **P < 0.01 versus normal control group; ^#^P < 0.05 and ^##^P < 0.01 versus ALI 6 h group.

W/D weight ratio of the lung and total protein concentration in BALF are markers of vascular permeability. As shown in Fig. 2a, W/D weight ratio of the lung was significantly higher in ALI 6 h group than that in normal control group (Con group: 4.4 ± 0.1, ALI 6 h group: 4.9 ± 0.2, P = 0.018, Fig. 2a). W/D weight ratio of the lung was significantly higher in ALI 24 h group than that in normal control group (Con group: 4.4 ± 0.1, ALI 24 h group: 5.3 ± 0.3, P = 0.014, Fig. 2a). W/D weight ratio of the lung was higher in ALI 24 h group than that in ALI 6 h group (ALI 6 h group: 4.9 ± 0.2, ALI 24 h group: 5.3 ± 0.3, P = 0.23, Fig. 2a).

As shown in Fig. 2b, total protein concentration in BALF was significantly higher in ALI 6 h group than that in normal control group (Con group: 0.3 ± 0.04, ALI 6 h group: 1.8 ± 0.2, P = 0.0004, Fig. 2b). Total protein concentration in BALF was significantly higher in ALI 24 h group than that in normal control group (Con group: 0.3 ± 0.04, ALI 24 h group: 2.1 ± 0.2, P < 0.0001, Fig. 2b). Total protein concentration in BALF was higher in ALI 24 h group than that in ALI 6 h group (Con group: 0.3 ± 0.04, ALI 6 h group: 1.8 ± 0.2, P = 0.14, Fig. 2b).

### MPO activity in lung homogenates

Activated neutrophils were implicated in the pathophysiology of smoke inhalation injury^26^. MPO is an indicator of neutrophil accumulation. As shown in Fig. 2c, MPO activity was significantly higher in ALI 6 h group than that in normal control group (Con group: 0.26 ± 0.02 U/g, ALI 6 h group: 1.0 ± 0.1 U/g, P = 0.0004, Fig. 2c). MPO activity was significantly higher in ALI 24 h group than that in normal control group (Con group: 0.26 ± 0.02 U/g, ALI 24 h group: 2.1 ± 0.2 U/g, P < 0.0001, Fig. 2c). MPO activity was significantly higher in ALI 24 h group than that in ALI 6 h group (ALI 6 h group: 1.0 ± 0.1 U/g, ALI 24 h group: 2.1 ± 0.2 U/g, P = 0.0009, Fig. 2c).

### Histopathology

Pulmonary histopathological changes were evaluated by H&E staining. As illustrated in Fig. 2d, no destructive changes were observed in lung tissues of rats from normal control group. At 6 h post-smoke inhalation, the lung tissues of rats exhibited interstitial infiltration of inflammatory cells, hemorrhage in alveolar and edema. At 24 h post-smoke inhalation, the lung tissues of rats showed notable pro-inflammatory alterations characterized by obvious thickening of the alveolar wall (interstitial edema), diffuse hemorrhage in the lung tissue and infiltration of inflammatory cells into interstitial tissue and alveolar space.

### Total and differential white blood cell counts in BALF

To assess the effect of smoke inhalation on lung inflammation, BALF was collected from rats in smoke inhalation and control group. As illustrated in Fig. 2f, total number of cells in BALF was dramatically higher in ALI 6 h group than that in normal control group (Con group: 1.3 ± 0. 2 × 10^5^/ml, ALI 6 h group: 15.9 ± 2.0 × 10^5^/ml, P < 0.0001, Fig. 2f). Total number of cells in BALF was dramatically higher in ALI 24 h group than that in normal control group (Con group: 1.3 ± 0. 2 × 10^5^/ml, ALI 24 h group: 24.1 ± 1.6 × 10^5^/ml, P = 0.002, Fig. 2f). Total number of cells in BALF was dramatically higher in ALI 24 h group than that in ALI 6 h group (ALI 6 h group: 15.9 ± 2.0 × 10^5^/ml, ALI 24 h group: 24.1 ± 1.6 × 10^5^/ml, P = 0.005, Fig. 2f).

To further evaluate the effect of smoke inhalation on leukocyte populations, differential cell count was performed. As expected, there was a marked increase of all inflammatory cells in BALF after smoke inhalation.

The number of neutrophils was seven times higher in ALI 6 h group than that in normal control group (Con group: 0.01 ± 0. 002 × 10^5^/ml, ALI 6 h group: 7.5 ± 0.5 × 10^5^/ml, P < 0.0001, Fig. 2f). The number of neutrophils was thirteen times higher in ALI 24 h group than that in normal control group (Con group: 0.01 ± 0. 002 × 10^5^/ml, ALI 24 h group: 13.9 ± 1.0 × 10^5^/ml, P = 0.0005, Fig. 2f). The number of neutrophils was two times higher in ALI 24 h group than that in ALI 6 h group (ALI 6 h group: 7.5 ± 0.5 × 10^5^/ml, ALI 24 h group: 13.9 ± 1.0 × 10^5^/ml, P = 0.0006, Fig. 2f).

The number of lymphocytes was three times higher in ALI 6 h group than that in normal control group (Con group: 0.07 ± 0. 02 × 10^5^/ml, ALI 6 h group: 3.1 ± 0.1 × 10^5^/ml, P < 0.0001, Fig. 2f). The number of lymphocytes was seven times higher in ALI 24 h group than that in normal control group (Con group: 0.07 ± 0. 02 × 10^5^/ml, ALI 24 h group: 6.9 ± 0.4 × 10^5^/ml, P = 0.0012, Fig. 2f). The number of lymphocytes was two times higher in ALI 24 h group than that in ALI 6 h group (ALI 6 h group: 3.1 ± 0.1 × 10^5^/ml, ALI 24 h group: 6.9 ± 0.4 × 10^5^/ml, P < 0.0001, Fig. 2f).

The number of macrophages was five times higher in ALI 6 h group than that in normal control group (Con group: 1.1 ± 0.3 × 10^5^/ml, ALI 6 h group: 4.9 ± 0.3 × 10^5^/ml, P = 0.0002, Fig. 2f). The number of macrophages was three times higher in ALI 24 h group than that in normal control group (Con group: 1.1 ± 0.3 × 10^5^/ml, ALI 24 h group: 3.7 ± 0.2 × 10^5^/ml, P = 0.0002, Fig. 2f). However, the number of macrophages was significantly lower in ALI 24 h group than that in ALI 6 h group (ALI 6 h group: 4.9 ± 0.3 × 10^5^/ml, ALI 24 h group: 3.7 ± 0.2 × 10^5^/ml, P = 0.0039, Fig. 2f).

### Expression of CD4^+^ T cells in peripheral blood and lung

**Figure 3:**
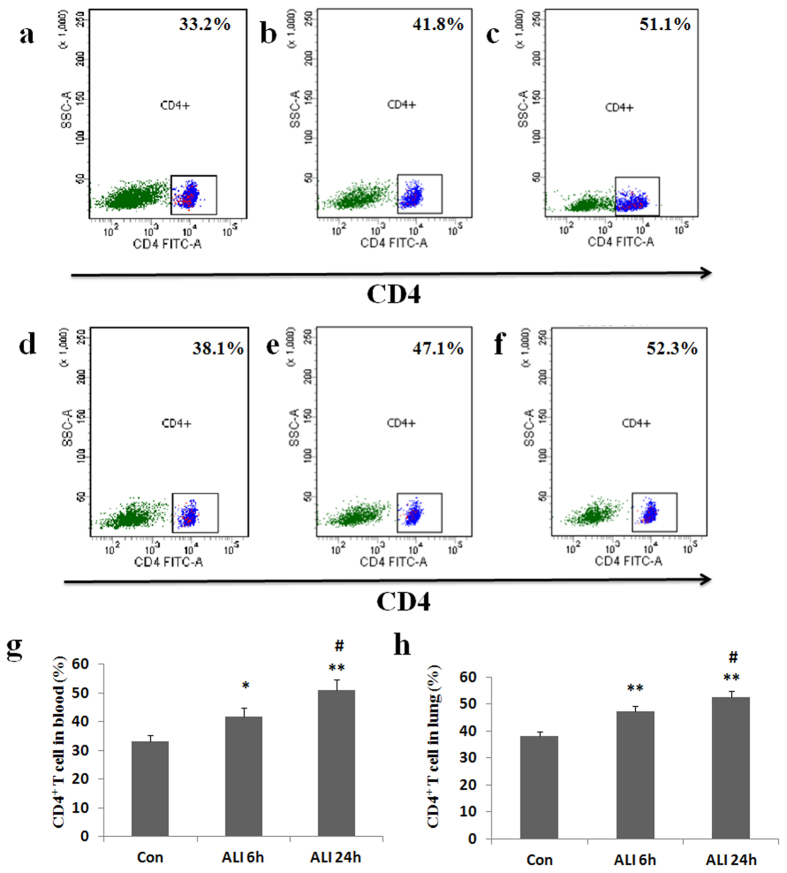
Expression of CD4^+^ T cells. Lymphocytes were surface-stained with CD4 antibody, and analyzed by flow cytometry. (**a**) CD4^+^ T subsets were gated by flow cytometry in Con group in peripheral blood; (**b**) CD4^+^ T subsets were gated by flow cytometry in ALI 6 h group in peripheral blood; (**c**) CD4^+^ T subsets were gated by flow cytometry in ALI 24 h group in peripheral blood; (**d**) CD4^+^ T subsets were gated by flow cytometry in Con group in lung; (**e**) CD4^+^ T subsets were gated by flow cytometry in ALI 6 h group in lung; (**f**) CD4^+^ T subsets were gated by flow cytometry in ALI 24 h group in lung; (**g**) The percentage of CD4^+^ T cells in peripheral blood were presented as mean ± S.E.M; (**h**) The percentage of CD4^+^ T cells in lung were presented as mean ± S.E.M. Con (normal control group, ambient air inhalation, n = 10), ALI 6 h (ALI group, smoke inhalation for 6 h, n = 10), ALI 24 h (ALI group, smoke inhalation for 24 h, n = 10). *P < 0.05 and **P < 0.01 versus normal control group; ^#^P < 0.05 and ^##^P < 0.01 versus ALI 6 h group.

To assess the effect of smoke inhalation on CD4^+^ T cell expression, peripheral blood and lung samples were collected for flow cytometry analysis. CD4^+^ T cells were gated. As shown in Fig. 3, the percentage of CD4^+^ T cells in peripheral blood was significantly higher in ALI 6 h group than that in normal control group (Con group: 33.2 ± 1.9%, ALI 6 h group: 41.8 ± 3.0%, P = 0.034, Fig. 3). The percentage of CD4^+^ T cells in peripheral blood was significantly higher in ALI 24 h group than that in normal control group (Con group: 33.2 ± 1.9%, ALI 24 h group: 51.1 ± 3.5%, P = 0.002, Fig. 3). The percentage of CD4^+^ T cells in peripheral blood was significantly higher in ALI 24 h group than that in ALI 6 h group (ALI 6 h group: 41.8 ± 3.0%, ALI 24 h group: 51.1 ± 3.5%, P = 0.02, Fig. 3).

Similarly, the percentage of CD4^+^ T cells in lung was significantly higher in ALI 6 h group than that in normal control group (Con group: 38.1 ± 1.6%, ALI 6 h group: 47.1 ± 2.1%, P = 0.004, Fig. 3). The percentage of CD4^+^ T cells in lung was significantly higher in ALI 24 h group than that in normal control group (Con group: 38.1 ± 1.6%, ALI 24 h group: 52.3 ± 2.3%, P = 0.0009, Fig. 3). The percentage of CD4^+^ T cells in lung was significantly higher in ALI 24 h group than that in ALI 6 h group (ALI 6 h group: 47.1 ± 2.1%, ALI 24 h group: 52.3 ± 2.3%, P = 0.044, Fig. 3).

### Th17 prevalence in peripheral blood and lung

To assess the effect of smoke inhalation on Th17 cell expansion, peripheral blood and lung samples were collected for flow cytometry analysis. Because CD4 marker of lymphocytes in blood of rats can be down-regulated by PMA stimulation, CD3^+^ CD8^−^ cells were gated and determined as CD4^+^ T cells. Prevalence of Th17 cells refers to the ratio of CD4^+^ IL-17A^+^ cells to the total amount of CD4^+^ T lymphocytes.

**Figure 4:**
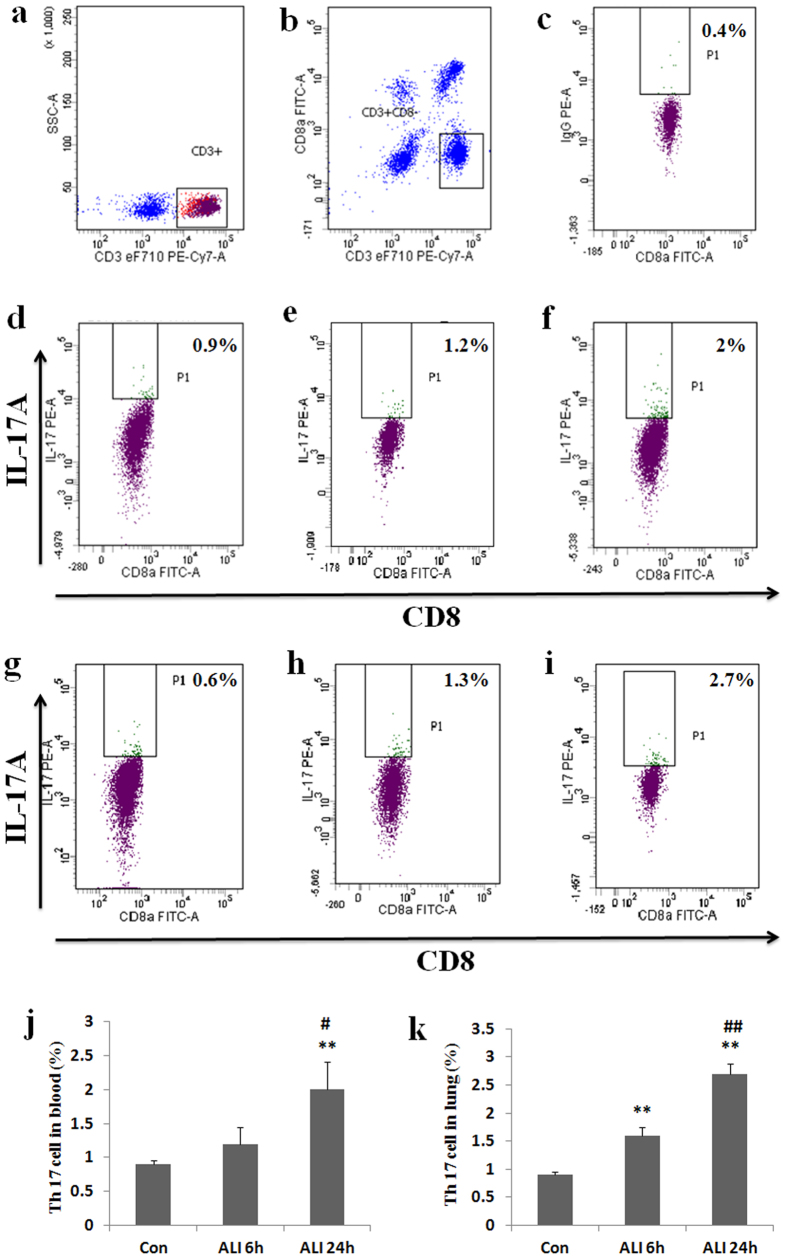
Th17 prevalence increased in rats with smoke inhalation. Lymphocytes were stimulated with PMA, ionomycin and BFA for 5 h, and then stained with labeled antibodies as described in Materials and methods. (**a**) T lymphocytes were gated by flow cytometry; (**b**) CD4^+^ (CD3^+^ CD8^−^) T subsets were gated by flow cytometry; (**c**) Isotype control; (**d**) IL-17A expression in CD4^+^ T cells were gated by flow cytometry in Con group in peripheral blood; (**e**) IL-17A expression in CD4^+^ T cells were gated by flow cytometry in ALI 6 h group in peripheral blood; (**f**) IL-17A expression in CD4^+^ T cells were gated by flow cytometry in ALI 24 h group in peripheral blood; (**g**) IL-17A expression in CD4^+^ T cells were gated by flow cytometry in Con group in lung; (**h**) IL-17A expression in CD4^+^ T cells were gated by flow cytometry in ALI 6 h group in lung; (**i**) IL-17A expression in CD4^+^ T cells were gated by flow cytometry in ALI 24 h group in lung; (**j**) The circulating Th17 cell frequency was presented as mean ± S.E.M; (**k**) The Th17 cell frequency in lung was presented as mean ± S.E.M. Con (normal control group, ambient air inhalation, n = 10), ALI 6 h (ALI group, smoke inhalation for 6 h, n = 10), ALI 24 h (ALI group, smoke inhalation for 24 h, n = 10). *P < 0.05 and **P < 0.01 versus normal control group; ^#^P < 0.05 and ^##^P < 0.01 versus ALI 6 h group.

As shown in Fig. 4, the prevalence of Th17 (CD4^+^ IL-17A^+^/CD4^+^ T cells) in peripheral blood was higher in ALI 6 h group than that in normal control group (Con group: 0.9 ± 0.06%, ALI 6 h group: 1.2 ± 0.25%, P = 0.07, Fig. 4). The prevalence of Th17 (CD4^+^ IL-17A^+^/CD4^+^ T cells) in peripheral blood was significantly higher in ALI 24 h group than that in normal control group (Con group: 0.9 ± 0.06%, ALI 24 h group: 2 ± 0.4%, P = 0.004, Fig. 4). The prevalence of Th17 (CD4^+^ IL-17A^+^/CD4^+^ T cells) in peripheral blood was significantly higher in ALI 24 h group than that in ALI 6 h group (ALI 6 h group: 1.2 ± 0.25%, ALI 24 h group: 2 ± 0.4%, P = 0.022, Fig. 4).

Similarly, the prevalence of Th17 (CD4^+^ IL-17A^+^/CD4^+^ T cells) in lung was significantly higher in ALI 6 h group than that in normal control group (Con group: 0.8 ± 0.09%, ALI 6 h group: 1.6 ± 0.15%, P = 0.003, Fig. 4). The prevalence of Th17 (CD4^+^ IL-17A^+^/CD4^+^ T cells) in lung was significantly higher in ALI 24 h group than that in normal control group (Con group: 0.8 ± 0.09%, ALI 24 h group: 2.7 ± 0.17%, P < 0.0001, Fig. 4). The prevalence of Th17 (CD4^+^ IL-17A^+^/CD4^+^ T cells) in lung was significantly higher in ALI 24 h group than that in ALI 6 h group (ALI 6 h group: 1.6 ± 0.15%, ALI 24 h group: 2.7 ± 0.17%, P = 0.0006, Fig. 4).

### CD4^+^ CD25^+^ Foxp3^+^ Treg prevalence in peripheral blood and lung

**Figure 5:**
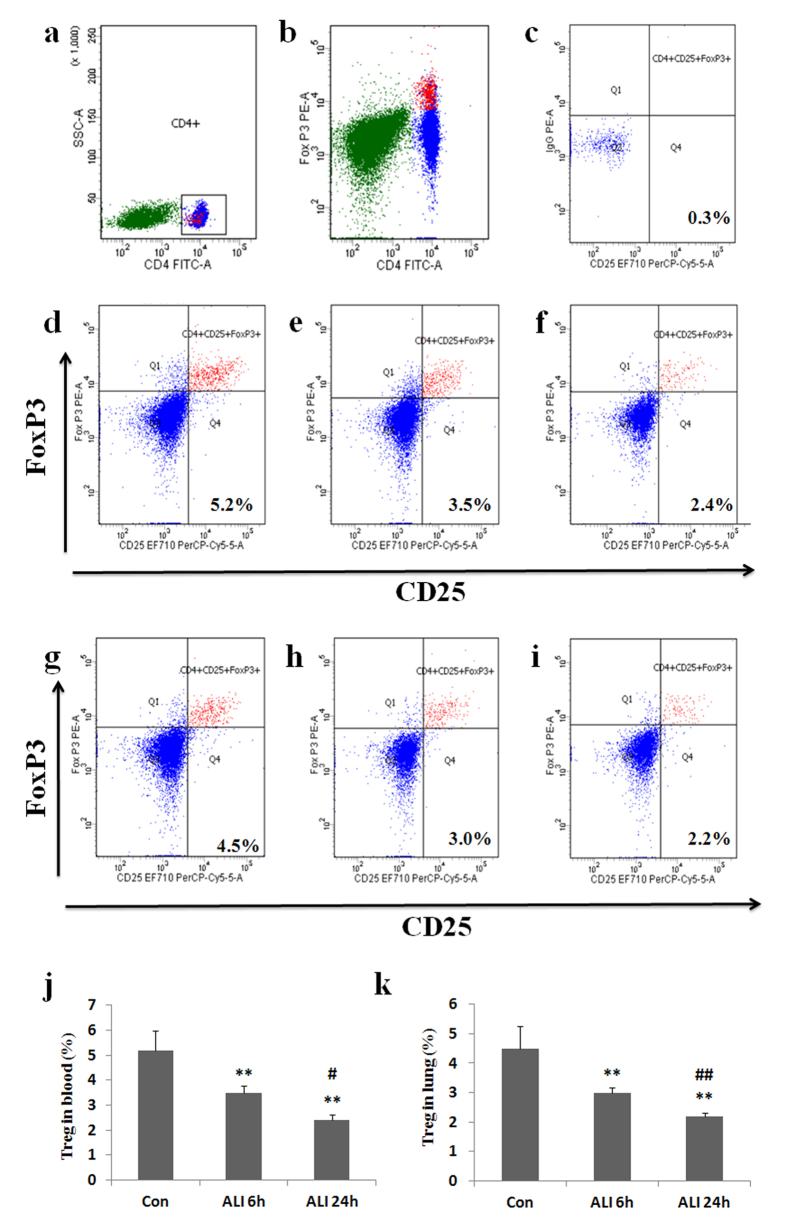
CD4^+^ CD25^+^ Foxp3^+^ Treg prevalence decreased in rats with smoke inhalation. Lymphocytes were stained with labeled antibodies as described in Materials and methods. (**a**) CD4^+^ T subsets were gated by flow cytometry; (**b**) Foxp3 expression in CD4^+^ T cells were gated by flow cytometry; (**c**) Isotype control; (**d**) CD25 and Foxp3 expression in CD4^+^ T subsets were gated by flow cytometry in Con group in peripheral blood; (**e**) CD25 and Foxp3 expression in CD4^+^ T subsets were gated by flow cytometry in ALI 6 h group in peripheral blood; (**f**) CD25 and Foxp3 expression in CD4^+^ T subsets were gated by flow cytometry in ALI 24 h group in peripheral blood; (**g**) CD25 and Foxp3 expression in CD4^+^ T subsets were gated by flow cytometry in Con group in lung; (**h**) CD25 and Foxp3 expression in CD4^+^ T subsets were gated by flow cytometry in ALI 6 h group in lung; (**i**) CD25 and Foxp3 expression in CD4^+^ T subsets were gated by flow cytometry in ALI 24 h group in lung; (**j**) The circulating Treg frequency was presented as mean ± S.E.M; (**k**) The Treg frequency in lung was presented as mean ± S.E.M. Con (normal control group, ambient air inhalation, n = 10), ALI 6 h (ALI group, smoke inhalation for 6 h, n = 10), ALI 24 h (ALI group, smoke inhalation for 24 h, n = 10). *P < 0.05 and **P < 0.01 versus normal control group; ^#^P < 0.05 and ^##^P < 0.01 versus ALI 6 h group.

Treg prevalence was also analyzed by flow cytometry. Prevalence of Treg refers to the ratio of CD4^+^ CD25^+^ Foxp3^+^ cells to the total amount of CD4^+^ T lymphocytes. As shown in Fig. 5, the prevalence of Treg (CD4^+^ CD25^+^ Foxp3^+^/CD4^+^ T cells) in peripheral blood was markedly lower in ALI 6 h group than that in normal control group (Con group: 5.2 ± 0.8%, ALI 6 h group: 3.5 ± 0.3%, P = 0.0045, Fig. 5). The prevalence of Treg (CD4^+^ CD25^+^ Foxp3^+^/CD4^+^ T cells) in peripheral blood was markedly lower in ALI 24 h group than that in normal control group (Con group: 5.2 ± 0.8%, ALI 24 h group: 2.4 ± 0.3%, P = 0.0022, Fig. 5). The prevalence of Treg (CD4^+^ CD25^+^ Foxp3^+^/CD4^+^ T cells) in peripheral blood was markedly lower in ALI 24 h group than that in ALI 6 h group (ALI 6 h group: 3.5 ± 0.3%, ALI 24 h group: 2.4 ± 0.3%, P = 0.014, Fig. 5).

Similarly, the prevalence of Treg (CD4^+^ CD25^+^ Foxp3^+^/CD4^+^ T cells) in lung was markedly lower in ALI 6 h group than that in normal control group (Con group: 4.5 ± 0.7%, ALI 6 h group: 3.0 ± 0.2%, P = 0.0069, Fig. 5). The prevalence of Treg (CD4^+^ CD25^+^ Foxp3^+^/CD4^+^ T cells) in lung was markedly lower in ALI 24 h group than that in normal control group (Con group: 4.5 ± 0.7%, ALI 24 h group: 2.2 ± 0.1%, P = 0.0029, Fig. 5). The prevalence of Treg (CD4^+^ CD25^+^ Foxp3^+^/CD4^+^ T cells) in lung was markedly lower in ALI 24 h group than that in ALI 6 h group (ALI 6 h group: 3.0 ± 0.2%, ALI 24 h group: 2.2 ± 0.1%, P = 0.0075, Fig. 5).

### Th17/Treg imbalance in peripheral blood and lung

**Figure 6:**
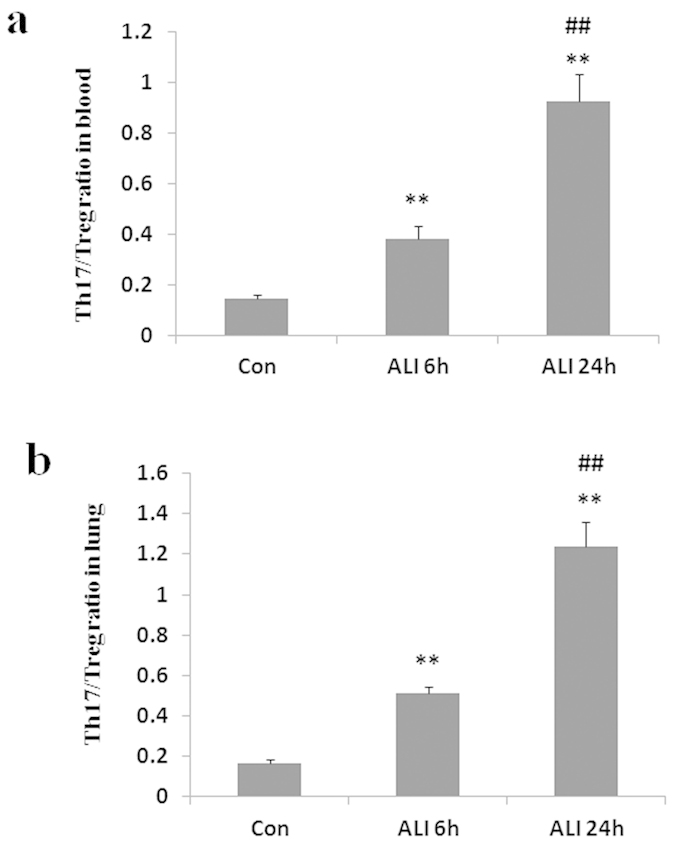
The ratio of Th17/Treg. There was a significant increase in the ratio of Th17/Treg in peripheral blood and lung from rats with smoke inhalation. (**a**) The ratio of Th17/Treg in peripheral blood; (**b**) The ratio of Th17/Treg in lung. Con (normal control group, ambient air inhalation, n = 10), ALI 6 h (ALI group, smoke inhalation for 6 h, n = 10), ALI 24 h (ALI group, smoke inhalation for 24 h, n = 10); data are presented as mean ± S.E.M. *P < 0.05 and **P < 0.01 versus normal control group; ^#^P < 0.05 and ^##^P < 0.01 versus ALI 6 h group.

We observed that a remarkable imbalance of the mean ratio of Th17 to Treg existed after smoke inhalation. As shown in Fig. 6, the ratio of Th17/Treg in peripheral blood was significantly elevated in ALI 6 h group than that in normal control group (Con group: 0.1 ± 0.01, ALI 6 h group: 0.4 ± 0.05, P = 0.0011, Fig. 6). The ratio of Th17/Treg in peripheral blood was significantly elevated in ALI 24 h group than that in normal control group (Con group: 0.1 ± 0.01, ALI 24 h group: 0.9 ± 0.1, P = 0.0002, Fig. 6). The ratio of Th17/Treg in peripheral blood was significantly elevated in ALI 24 h group than that in ALI 6 h group (ALI 6 h group: 0.4 ± 0.05, ALI 24 h group: 0.9 ± 0.1, P = 0.0012, Fig. 6).

Similarly, the ratio of Th17/Treg in lung was significantly elevated in ALI 6 h group than that in normal control group (Con group: 0.2 ± 0.02, ALI 6 h group: 0.5 ± 0.03, P < 0.0001, Fig. 6). The ratio of Th17/Treg in lung was significantly elevated in ALI 24 h group than that in normal control group (Con group: 0.2 ± 0.02, ALI 24 h group: 1.2 ± 0.1, P = 0.0001, Fig. 6). The ratio of Th17/Treg in lung was significantly elevated in ALI 24 h group than that in ALI 6 h group (ALI 6 h group: 0.5 ± 0.03, ALI 24 h group: 1.2 ± 0.1, P = 0.0005, Fig. 6).

### Th1 prevalence in peripheral blood and lung

To assess the effect of smoke inhalation on Th1 cell expansion, peripheral blood and lung samples were collected for flow cytometry analysis. Prevalence of Th1 refers to the ratio of CD4^+^ IFN-γ^+^ cells to the total amount of CD4^+^ T lymphocytes.

As shown in Supplementary Fig. S1, the prevalence of Th1 (CD4^+^ IFN-γ^+^/CD4^+^ T cells) in peripheral blood was markedly lower in ALI 6 h group than that in normal control group (Con group: 2.6 ± 0.4%, ALI 6 h group: 1.6 ± 0.15%, P = 0.014, Supplementary Fig. S1). The prevalence of Th1 (CD4^+^ IFN-γ^+^/CD4^+^ T cells) in peripheral blood was markedly lower in ALI 24 h group than that in normal control group (Con group: 2.6 ± 0.4%, ALI 24 h group: 0.8 ± 0.15%, P = 0.002, Supplementary Fig. S1). The prevalence of Th1 (CD4^+^ IFN-γ^+^/CD4^+^ T cells) in peripheral blood was markedly lower in ALI 24 h group than that in ALI 6 h group (ALI 6 h group: 1.6 ± 0.15%, ALI 24 h group: 0.8 ± 0.15%, P = 0.003, Supplementary Fig. S1).

Similarly, the prevalence of Th1 (CD4^+^ IFN-γ^+^/CD4^+^ T cells) in lung was markedly lower in ALI 6 h group than that in normal control group (Con group: 3.7 ± 0.35%, ALI 6 h group: 2.1 ± 0.2%, P = 0.003, Supplementary Fig. S1). The prevalence of Th1 (CD4^+^ IFN-γ^+^/CD4^+^ T cells) in lung was markedly lower in ALI 24 h group than that in normal control group (Con group: 3.7 ± 0.35%, ALI 24 h group: 1.0 ± 0.12%, P = 0.0003, Supplementary Fig. S1). The prevalence of Th1 (CD4^+^ IFN-γ^+^/CD4^+^ T cells) in lung was markedly lower in ALI 24 h group than that in ALI 6 h group (ALI 6 h group: 2.1 ± 0.2%, ALI 24 h group: 1.0 ± 0.12%, P = 0.0013, Supplementary Fig. S1).

### Th2 prevalence in peripheral blood and lung

To assess the effect of smoke inhalation on Th2 cell expansion, peripheral blood and lung samples were collected for flow cytometry analysis. Prevalence of Th2 refers to the ratio of CD4^+^ IL-4^+^ cells to the total amount of CD4^+^ T lymphocytes.

As shown in Supplementary Fig. S2, the prevalence of Th2 (CD4^+^ IL-4^+^/CD4^+^ T cells) in peripheral blood was significantly higher in ALI 6 h group than that in normal control group (Con group: 6.8 ± 1.3%, ALI 6 h group: 10.2 ± 0.9%, P = 0.017, Supplementary Fig. S2). The prevalence of Th2 (CD4^+^ IL-4^+^/CD4^+^ T cells) in peripheral blood was significantly higher in ALI 24 h group than that in normal control group (Con group: 6.8 ± 1.3%, ALI 24 h group: 12 ± 0.68%, P = 0.0037, Supplementary Fig. S2). The prevalence of Th2 (CD4^+^ IL-4^+^/CD4^+^ T cells) in peripheral blood was higher in ALI 24 h group than that in ALI 6 h group (ALI 6 h group: 10.2 ± 0.9%, ALI 24 h group: 12 ± 0.68%, P = 0.067, Supplementary Fig. S2).

Similarly, the prevalence of Th2 (CD4^+^ IL-4^+^/CD4^+^ T cells) in lung was significantly higher in ALI 6 h group than that in normal control group (Con group: 9.8 ± 0.64%, ALI 6 h group: 12.3 ± 0.85%, P = 0.026, Supplementary Fig. S2). The prevalence of Th2 (CD4^+^ IL-4^+^/CD4^+^ T cells) in lung was significantly higher in ALI 24 h group than that in normal control group (Con group: 9.8 ± 0.64%, ALI 24 h group: 14.7 ± 0.47%, P = 0.0005, Supplementary Fig. S2). The prevalence of Th2 (CD4^+^ IL-4^+^/CD4^+^ T cells) in lung was significantly higher in ALI 24 h group than that in ALI 6 h group (ALI 6 h group: 12.3 ± 0.85%, ALI 24 h group: 14.7 ± 0.47%, P = 0.009, Supplementary Fig. S2).

### Th1/Th2 imbalance in peripheral blood and lung

We observed that a remarkable imbalance of the mean ratio of Th1 to Th2 existed after smoke inhalation. As shown in Supplementary Fig. S3, the ratio of Th1/Th2 in peripheral blood was significantly lower in ALI 6 h group than that in normal control group (Con group: 0.36 ± 0.02, ALI 6 h group: 0.14 ± 0.01, P = 0.0001, Supplementary Fig. S3). The ratio of Th1/Th2 in peripheral blood was significantly lower in ALI 24 h group than that in normal control group (Con group: 0.36 ± 0.02, ALI 24 h group: 0.06 ± 0.01, P < 0.0001, Supplementary Fig. S3). The ratio of Th1/Th2 in peripheral blood was significantly lower in ALI 24 h group than that in ALI 6 h group (ALI 6 h group: 0.14 ± 0.01, ALI 24 h group: 0.06 ± 0.01, P = 0.0019, Supplementary Fig. S3).

Similarly, the ratio of Th1/Th2 in lung was significantly lower in ALI 6 h group than that in normal control group (Con group: 0.37 ± 0.01, ALI 6 h group: 0.17 ± 0.01, P < 0.0001, Supplementary Fig. S3). The ratio of Th1/Th2 in lung was significantly lower in ALI 24 h group than that in normal control group (Con group: 0.37 ± 0.01, ALI 24 h group: 0.06 ± 0.01, P < 0.0001, Supplementary Fig. S3). The ratio of Th1/Th2 in lung was significantly lower in ALI 24 h group than that in ALI 6 h group (ALI 6 h group: 0.17 ± 0.01, ALI 24 h group: 0.06 ± 0.01, P < 0.0001, Supplementary Fig. S3).

### Th17- and Treg-related cytokines in serum and BALF supernatant

In order to determine the changes of Th17- and Treg-related cytokine profile, serum and BALF supernatant from rats were collected. Th17-related cytokines (IL-6, IL-17A, TGF-β and IL-23) and Treg-related cytokines (IL-10, IL-2 and IL-35) were measured by enzyme-linked immunosorbent assay (ELISA; R&D Systems, USA) and Luminex technology (Luminex, USA) according to the manufacturer’s instructions.

As demonstrated in Table 2, the levels of Th17-related cytokines (IL-6, IL-17A, TGF-β and IL-23) in serum were significantly higher in ALI 6 h group than that in normal control group. The levels of Th17-related cytokines (IL-6, IL-17A, TGF-β and IL-23) in serum were significantly higher in ALI 24 h group than that in normal control group and ALI 6 h group.

In contrast, the levels of Treg-related cytokines (IL-10, IL-2 and IL-35) in serum were significantly decreased in ALI 6 h group than that in normal control group (Table 3). The levels of Treg-related cytokines (IL-10, IL-2 and IL-35) in serum were significantly decreased in ALI 24 h group than that in normal control group and ALI 6 h group (Table 3).

In BALF supernatant, the levels of Th17-related cytokines (IL-6, IL-17A, TGF-β and IL-23, Table 4) and Treg-related cytokines (IL-10, IL-2, IL-35, Table 5) also showed the same change trends as those in the serum. The results were consistent with the increased prevalence of Th17 and decreased Tregs in rats.

### Th1- and Th2-related cytokines in serum and BALF supernatant

In order to determine the changes of Th1- and Th2-related cytokine profile, serum and BALF supernatant from rats were collected. Th1-related cytokine (IFN-γ) and Th2-related cytokine (IL-4) were determined by Luminex technology according to the manufacturer’s instructions.

As demonstrated in Supplementary Table S1, the level of Th1-related cytokine (IFN-γ) in serum was significantly decreased in ALI 6 h group than that in normal control group. The level of Th1-related cytokine (IFN-γ) in serum was significantly decreased in ALI 24 h group than that in normal control group and ALI 6 h group.

In contrast, the level of Th2-related cytokine (IL-4) in serum was significantly higher in ALI 6 h group than that in normal control group (Supplementary Table S1). The level of Th2-related cytokine (IL-4) in serum was significantly higher in ALI 24 h group than that in normal control group and ALI 6 h group (Supplementary Table S1).

In BALF supernatant, the levels of Th1-related cytokine (IFN-γ, Supplementary Table S2) and Th2-related cytokine (IL-4, Supplementary Table S2) also showed the same change trends as those in the serum. The results were consistent with the increased prevalence of Th2 and decreased Th1 cells in rats.

## Discussion

Despite decades of intense research, the molecular mechanisms involved in the pathogenesis of smoke inhalation-induced acute lung injury are poorly defined. Traditionally, attention has been focused on the roles of neutrophils and macrophages in smoke inhalation-induced acute lung injury. However, the role of adaptive immune cells in this disease is less well defined. It has been suggested that T lymphocytes, especially CD4^+^ T cells, contribute to the progression of autoimmune and inflammatory diseases^13^. Recently, lymphocytes have drawn increased attention, and there is accumulating evidence indicating that CD4^+^ T lymphocytes play important roles in the development and progression of ALI/ARDS. Therefore, we aimd to investigate changes of lymphocytes, in particular the balance of Th17/Treg and related cytokine profile involved in the immune pathogenesis of smoke inhalation-induced acute lung injury.

To explore the effect of smoke inhalation on Th17/Treg balance, we used the self-made smoke generator to induce the model of gunpowder smog-induced acute lung injury in rats. We found that mortality of rats increased with the addition of gunpowder dosage and prolongation of exposure time, and the exposure to the smoke generated by 10 g gunpowder for 8 min was the optimum condition. Therefore, the rats in smoke inhalation group were exposed to the smoke generated by 10 g gunpowder for 8 min in the present study.

We reported here an increase of Th17 cells and Th17-related cytokines (IL-17A, IL-6, IL-23 and TGF-β) in rats after smoke inhalation. On the other hand, the number of Tregs and Treg-related cytokines (IL-10, IL-2 and IL-35) in the peripheral blood of rats with smoke inhalation were decreased. These results suggest that the imbalance of Th17/Treg may be involved in the pathogenesis of smoke inhalation-induced acute lung injury.

CD4^+^ CD25^+^ Foxp3^+^ Treg is a critical sub-population of CD4^+^ T cells that is essential for maintaining self tolerance and preventing autoimmunity, for limiting chronic inflammatory diseases, such as asthma and inflammatory bowel disease, and for regulating homeostatic lymphocyte expansion^27^. A recent report by D’Alessio and coauthors^15^ investigated the role of lymphocytes in lipopolysaccharide-induced ALI. Their study showed that Treg depletion in wild-type mice delayed recovery, whereas Treg transfer into lymphocyte-deficient mice corrected the elevated levels of alveolar proinflammatory cytokines and increased the diminished transforming growth factor (TGF)-β and neutrophil apoptosis. In this study, we observed that CD4^+^ CD25^+^ Foxp3^+^ Treg in peripheral blood was markedly decreased at 24 h after smoke inhalation. Our result is consistent with a recent study demonstrating that mice with chronic cigarette smoke exposure showed significant decrease in Treg prevalence in peripheral blood and Treg-related cytokine such as IL-10^28^. Generally, Tregs are supposed to play an anti-inflammatory role mainly by contact-dependent suppression, consumption of limiting T cell growth factors such as IL-2 or releasing inhibitory cytokines such as IL-10 and TGF-β on other immune cells, including CD4^+^ T cells^13^,^29^. Similarly, we found that CD4^+^ T cells were increased in peripheral blood of rats in response to smoke inhalation, which is consistent with lower percentage of Tregs. Naïve CD4^+^ T cells differentiate into distinct functional subsets according to the local cytokine environment. The development of both Tregs and Th17 cells requires TGF-β, whereas IL-2 and IL-6 are key regulators interfering with differentiation of Treg and Th17 from naive T cells^30^,^31^. TGF-β and IL-2 are essential for the development of Tregs^32^. IL-2 maintains Foxp3^+^ Tregs^33^ and inhibits the polarization of Th17 cells^34^. IL-6 suppresses Treg differentiation^35^; in contrast, Th17 differentiation is initiated by IL-6 together with TGF-β^36^. From our study, we found that gunpowder smog inhalation decreased IL-2 level in BALF and serum of rats. This result can explain the lower percentage of Tregs and the higher percentage of Th17 cells observed in rats with smoke inhalation. Moreover, Treg may attempt to resolve inflammation by producing IL-10, which is a potent anti-inflammatory cytokine that inhibits the synthesis of many inflammatory molecules such as tumor necrosis factor (TNF)-α and IL-1β^37^. As a novel identified inhibitory cytokine, IL-35 is specifically produced by Tregs and is required for maximal suppressive activity^38^. Our results showed that both IL-10 and IL-35 were decreased in BALF and serum of rats after smoke inhalation, which are consistent with lower percentage of Tregs after smoke inhalation. Notably, research showed that Th2 responses inhibit TGF-β-induced Foxp3 expression and the formation of Tregs^39^,^40^,^41^. We found that gunpowder smog induced the Th1/Th2 imbalance favoring a Th2 shift. Thus, the decreased Tregs in rats with smoke inhalation are partly attributed to decreased level of IL-2 and enhanced Th2 immunity. Inconsistent with our study, an earlier report has shown that Treg percentage in peripheral blood was significantly higher in patients with ARDS than in control group^21^. The difference may be largely due to different study groups, limited scale of ARDS subjects, the course of the disease and methods for detecting Treg. Indeed, ARDS is a heterogeneous syndrome that can occur as a result of multiple insults and diseases. Despite of a common end state, it is noteworthy that ARDS between pulmonary and extrapulmonary may differ in morphology and respiratory physiology as well as response to therapeutic interventions^42^.

As a more recently discovered effector subset of CD4^+^ T cells, Th17 cell plays a key role in defense against extracellular pathogens, and promotes many autoimmune inflammatory diseases^13^. A recent study showed that the peripheral circulating Th17 cells and their associated cytokines increased in patients with early ARDS and may conduce to ARDS^21^. In our study, Th17 cells in peripheral blood were increased in rats with smoke inhalation, implying the possibility that Th17-mediated immune response is involved in the pathogenesis of smoke inhalation-induced acute lung injury. We also found that the changes of IL-17A were consistent with the change tendency of Th17 cells. As a predominant product and key effector molecule of Th17 cells, IL-17A appears to play a pivotal role in promoting activation and recruitment of leukocyte such as neutrophils and macrophages at lung or airway inflammation^43^,^44^,^45^,^46^,^47^. In addition, IL-17-deficient mice have an impaired neutrophilic response to allergen^48^. It may partly explain the increase of neutrophils and macrophages in BALF from rats with smoke inhalation in our study. The differentiation and development of Th17 cell are determined by the microenvironment. TGF-β, IL-6, IL-23 and IL-1β are all involved in the differentiation of human Th17 cells but IL-1β is not needed in mouse^49^,^50^. TGF-β alone induces the differentiation into Tregs, but in an IL-1- and/or IL-6-rich inflammatory milieu, the Th17 generation is enhanced while the Tregs are suppressed^51^. In the present study, both TGF-β and IL-6 were increased in serum and BALF of rats with smoke inhalation, which may inhibit the generation of TGF-β-induced Tregs and promote a pro-inflammatory T-cell response predominated by Th17 cells. IL-23 appears to be essential to expand and maintain Th17 cells and plays a crucial role in the establishment and maintenance of inflammatory and autoimmune diseases. Research has shown that IL-23 was increased in patients with chronic obstructive pulmonary disease^52^. In this study, the concentration of IL-23 was increased in serum and BALF of rats with smoke inhalation. Taken together, gunpowder smog inhalation promotes pro-inflammatory cytokines such as IL-6, TGF-β and IL-23, thereby promoting differentiation, development and maintenance of Th17 cells.

Remarkably, Th17 and Treg not only share reciprocal development pathways but also exert opposite functions in the immune response. Moreover, certain proinflammatory milieu could convert differentiated Tregs into Th17 cells^51^. Thus, the balance between Th17 and Treg may be critical for maintenance of immune homeostasis. In fact, the balance between Th17 and Treg response has been suggested as a new paradigm for a number of inflammation and autoimmune diseases^13^. In the present study, the Th17/Treg ratio was markedly increased in peripheral blood of rats after smoke inhalation, indicating that the proinflammatory environment in rats with smoke inhalation was in favor of the generation of Th17 cells relative to Tregs. Moreover, a recent study revealed a previously unexpected role of adaptive immune responses that increase alveolar permeability in ARDS^53^. Similarly, our findings suggest that Th17/Treg imbalance contributes to increased alveolar epithelial permeability. Histological findings also showed that interstitial inflammation and lung injury were more severe in response to smoke inhalation. Taken together, these findings indicate that Th17/Treg imbalance in favor of a proinflammatory Th17-response is likely to be involved in the immune pathogenesis of smoke inhalation-induced acute lung injury. Further studies on the functional link of Th17/Treg imbalance to the disease pathogenesis using knock-out strategies or neutralizing antibodies may be beneficial for clinical management of smoke inhalation-induced acute lung injury that accounts for extensive morbidity and mortality.

In summary, we demonstrated that Th17 prevalence was increased but Treg prevalence was decreased in the early stage of disease process, thus leading to Th17/Treg imbalance in the rat model of gunpowder smog-induced acute lung injury. The imbalance may partly be due to the circulating and local cytokine microenvironment. Our results suggest a potential role of Th17/Treg imbalance and related cytokine profile changes in the development and progression of smoke inhalation-induced acute lung injury.

## Methods

### Animals

Male Wistar rats aged 8–10 weeks and weighing 180–200 g were supplied by the laboratory animal center of the PLA General Hospital (Beijing, China) and raised carefully in accordance with National Institutes of Health on animal care and the ethical guidelines. Rats were raised in pathogen-free cages and kept at the temperature of 20–25 °C and the relative humidity of 50–70%. All experimental procedures were approved by the Ethics Committee of the Medical College of the People’s Liberation Army (permit number: SCXK (Beijing) 2012-0001).

### A rat model of smoke inhalation-induced acute lung injury

The self-made smoke generator was improved on the basis of the smog-generation device from our laboratory^54^, which consists of an electromagnetic oven for smoke production and a closed smoke chamber that houses the smoke-exposed rats. The two chambers were connected, with an electric fan on the wall. There was an iron plate on the electromagnetic oven to put on gunpowder. A glass door in the smoke chamber was applied to monitor the situation of rats. On control panel, there was a button for controlling the fan to adjust the smoke intensity in the smoke chamber and a device for monitoring the temperature and humidity. Using the smoke generator, smoke was generated by 10 g of gunpowder. The electromagnetic heater was turned off as soon as the flame burned out. Two rats were exposed each time to the smoke for 8 min.

### Detection of smoke composition

The concentration of oxygen (O_2_), carbon monoxide (CO), and hydrogen sulfide (H_2_S) was detected using an EM-4 L gas detector (Australia New Meter Group, Hong Kong, China). The particulate matters (PM) were analyzed by aerodynamic particle size spectrometer (TSI, MN, USA).

### Experimental protocol

Thirty rats were equally randomized to three groups: Con (normal control group, ambient air inhalation, n = 10), ALI 6 h (ALI group, smoke inhalation for 6 h, n = 10), ALI 24 h (ALI group, smoke inhalation for 24 h, n = 10). The rats were sacrificed at 6 h and 24 h. All rats were anesthetized intraperitoneally with pentobarbital sodium (50 mg/kg; Sigma-Aldrich Corporation, St. Louis, MO, USA), which was dissolved in normal saline (NS, NaCl, 0.9%) at the concentration of 10 mg/ml. Blood samples were collected. After the left lung was isolated with a micro clamp tied off at the right bronchus, three sequential 2.5 ml of ice-cold normal saline was injected intratracheally into the left bronchus to collect bronchoalveolar lavage fluid (BALF). The right upper lobe of the lung was fixed in 10% formalin for histological examination and the right lower lobe was prepared for wet to dry (W/D) weight ratio measurement.

### BALF analysis

BALF was collected and centrifuged (1000 g for 10 min) at 4 °C to pellet the cells. The pellet was analyzed for total cell. Differential cell count was prepared using a cytospin and stained with Wright-Giemsa. The supernatant was stored at −80 °C for subsequent cytokine assay and protein concentration. Protein concentration was determined by bicinchoninic acid (BCA) protein assay kit (Novagen, USA) to assess lung vascular permeability.

### Lung W/D weight ratio

The right lower lobe of the lung was collected to calculate lung W/D weight ratio. The lung was weighed immediately after collection and at 48 h after being dried in a 60 °C oven. The W/D weight ratio was obtained by dividing the wet weight by the dried weight to assess lung vascular permeability.

### Histopathology

The right upper lobe of the lung was fixed in 10% formalin, dehydrated, embedded in paraffin and cut into sections of 6 μm. After deparaffinization, the tissues were stained with hematoxylin and eosin (H&E), and the morphological lesions and changes in lung tissues were observed under a light microscope. Ten fields per section were examined to determine morphological lesions and changes in lung tissues. A semiquantitative system was used according to a modified scoring system^55^. The total histological score ranged from 0 to 7, which represented the summed scores of atelectasis, alveolar and interstitial edema, pulmonary hemorrhage and interstitial infiltration of inflammatory cells.

### Lung tissue myeloperoxidase assay

Myeloperoxidase (MPO) activity in the lung parenchyma, a marker of neutrophil infiltration, was assessed by chromometry according to the manufacturer’s instructions (Nanjing Jiancheng Bioengineering Institute, Nanjing, China). Briefly, lung tissues (100 mg each) were homogenized in normal saline and centrifuged for 20 min at 10,000 g at 4 °C. An aliquot of 0.2 ml supernatant was added to react with 1.6 mM of tetramethyl-benzidine and 0.1 mM of H_2_O_2_. The rate of change in absorbance was measured by a spectrophotometer at 460 nm. MPO activity was expressed in units per gram weight of wet tissue.

### Flow cytometry analysis

For analysis of Tregs, blood and lung tissue samples were collected into tubes without stimulation of phorbol 12-myristate 13-acetate (PMA; ENZO Alexis-Biomol, USA) and ionomycin (ENZO Alexis-Biomol, USA) and surface-labeled with FITC-labeled anti-rat CD4 (eBioscience, San Diego, CA, USA) and PE-Cy5-labeled anti-rat CD25 antibodies (eBioscience, San Diego, CA, USA), followed by fixation and permeabilization and intracellular staining with PE-conjugated anti-rat Foxp3 antibody (eBioscience, San Diego, CA, USA). To determine the phenotypes of Th1/Th2/Th17 cells in whole blood and lung tissue of rats, 20 ng/mL PMA and 500 ng/mL ionomycin in the presence of 2.5 μg/mL brefeldin A (BFA; ENZO Alexis-Biomol, USA) were used to stimulate cytokine expression of Th1/Th2/Th17 cells for 5 h. CD3^+^ CD8^−^ T cells were used to delimitate CD4^+^ T cells as CD4 is down-modulated when cells are activated by PMA. The cells were surface-stained with PerCP-eF710-labeled anti-rat CD3 (eBioscience, San Diego, CA, USA) and fluorescein isothiocyanate (FITC)-labeled anti-rat CD8 antibodies (eBioscience, San Diego, CA, USA) for 30 min, and then fixed and permeabilized using Fix&Perm Reagent (Invitrogen, USA). Subsequently, phycoerythrin (PE)-conjugated anti-rat IL-17A antibody (eBioscience, San Diego, CA, USA), PE-conjugated anti-rat IFN-γ antibody (eBioscience, San Diego, CA, USA) and PE-conjugated anti-rat IL-4 antibody (eBioscience, San Diego, CA, USA) were used for intracellular cytokine staining. Isotype controls (eBioscience, San Diego, CA, USA) were treated to enable correct compensation and confirm antibody specificity. After washing procedures, the stained cells were analyzed by flow cytometry (BD FACSCanto™II, USA). The results were analyzed with CellQuest software (BD FACSDiva, USA), and were presented as a percentage of cytokine-producing cells in CD4^+^ T cells.

### Cytokine assay

The concentrations of Th1-related cytokine (IFN-γ), Th2-related cytokine (IL-4), Th17-related cytokines (IL-6, IL-17A, TGF-β and IL-23) and Treg-related cytokines (IL-10, IL-2 and IL-35) in serum and BALF supernatant were determined by enzyme-linked immunosorbent assay (ELISA; R&D Systems, USA) and Luminex technology (Luminex, USA) according to the manufacturer’s instructions.

### Statistics and software

Statistical Package for the Social Sciences (SPSS) statistical software for Windows (SPSS Inc., Chicago, USA, version 17.0) was applied for all statistical analysis. All data were expressed as mean ± standard error mean (S.E.M). Differences between groups were examined for statistical significance by Kruskal-Wallis *H* test. P < 0.05 was considered significant.

## Additional Information

**How to cite this article**: Zhang, F. *et al*. Imbalance of Th17/Tregs in rats with smoke inhalation-induced acute lung injury. *Sci. Rep.* **6**, 21348; doi: 10.1038/srep21348 (2016).

## Supplementary Material

Supplementary Information

## Figures and Tables

**Table 1:** Time course changes of smoke composition.

	O_2_ (%)	CO (ppm)	H_2_S (ppm)	T(°C)
3 min	18.9 ± 0.19	308 ± 16.13	0.55 ± 0.09	27.2 ± 0.93
8 min	18.6 ± 0.26	302 ± 14.75	0.52 ± 0.06	26.6 ± 1.59
10 min	18.7 ± 0.22	298 ± 9.52	0.47 ± 0.04	25.8 ± 1.21

O_2_, oxygen; CO, carbon monoxide; H_2_S, hydrogen sulfide; T, temperature.

**Table 2:** Th17-related cytokines in serum.

Cytokines	Con	ALI 6 h	ALI 24 h
IL-6 (pg/ml)	63.6 ± 11.6	122.3 ± 16.9**	192.0 ± 18.9**##
IL-17A (pg/ml)	6.1 ± 1.5	13.3 ± 2.1**	19.3 ± 1.9**#
TGF-β (pg/ml)	295.6 ± 41.4	481.7 ± 49.1*	769.1 ± 83.2**##
IL-23 (pg/ml)	116.8 ± 4.5	131.0 ± 2.6**	149.6 ± 2.3**##

Con (normal control group, ambient air inhalation, n = 10), ALI 6 h (ALI group, smoke inhalation for 6 h, n = 10), ALI 24 h (ALI group, smoke inhalation for 24 h, n = 10); data are presented as mean ± S.E.M. *P < 0.05 and **P < 0.01 versus normal control group; ^#^P < 0.05 and ^##^P < 0.01 versus ALI 6 h group.

**Table 3:** Treg-related cytokines in serum.

Cytokines	Con	ALI 6 h	ALI 24 h
IL-10 (pg/ml)	15.2 ± 2.5	10.4 ± 0.9*	6.1 ± 2.3**#
IL-2 (pg/ml)	21.5 ± 1.4	14.2 ± 1.3**	7.6 ± 1.2**#
IL-35 (pg/ml)	130.4 ± 0.5	119.3 ± 1.5**	112.1 ± 4.2**#

Con (normal control group, ambient air inhalation, n = 10), ALI 6 h (ALI group, smoke inhalation for 6 h, n = 10), ALI 24 h (ALI group, smoke inhalation for 24 h, n = 10); data are presented as mean ± S.E.M. *P < 0.05 and **P < 0.01 versus normal control group; ^#^P < 0.05 and ^##^P < 0.01 versus ALI 6 h group.

**Table 4:** Th17-related cytokines in BALF.

Cytokines	Con	ALI 6 h	ALI 24 h
IL-6 (pg/ml)	117.9 ± 14.1	427.7 ± 31.2**	666.9 ± 31.5**##
IL-17A (pg/ml)	3.5 ± 1.1	19.7 ± 5.0**	29.3 ± 3.2**#
TGF-β (pg/ml)	681.8 ± 21.4	897.0 ± 43.8*	1280.3 ± 160.7**##
IL-23 (pg/ml)	128.4 ± 2.3	137.6 ± 2.5**	141.5 ± 5.0*

Con (normal control group, ambient air inhalation, n = 10), ALI 6 h (ALI group, smoke inhalation for 6 h, n = 10), ALI 24 h (ALI group, smoke inhalation for 24 h, n = 10); data are presented as mean ± S.E.M. *P < 0.05 and **P < 0.01 versus normal control group; ^#^P < 0.05 and ^##^P < 0.01 versus ALI 6 h group.

**Table 5:** Treg-related cytokines in BALF.

Cytokines	Con	ALI 6 h	ALI 24 h
IL-10 (pg/ml)	23.0 ± 2.6	14.6 ± 0.9**	7.5 ± 1.2**##
IL-2 (pg/ml)	14.5 ± 3.6	7.7 ± 1.5*	3.1 ± 2.1**#
IL-35 (pg/ml)	159.8 ± 3.3	133.7 ± 6.4	109.5 ± 5.8**

Con (normal control group, ambient air inhalation, n = 10), ALI 6 h (ALI group, smoke inhalation for 6 h, n = 10), ALI 24 h (ALI group, smoke inhalation for 24 h, n = 10); data are presented as mean ± S.E.M. *P < 0.05 and **P < 0.01 versus normal control group; ^#^P < 0.05 and ^##^P < 0.01 versus ALI 6 h group.
