# Supplementary material for: Imbalance of Th17/Tregs in rats with smoke inhalation-induced acute lung injury
Source: Sci Rep. 2016 Feb 17;6:21348. doi: 10.1038/srep21348 (PMC4756332; doi:10.1038/srep21348)
Supplement: Supplementary file 1 — Supplementary Information [file 41598_2016_BFsrep21348_MOESM1_ESM.pdf]

Imbalance of Th17/Tregs in rats with smoke inhalation-induced acute lung injury

Fan Zhang, Mian-yang Li, Ya-ting Lan, Cheng-bin Wang *

Department of Clinical Laboratory Medicine, Chinese People's Liberation Army General Hospital & Postgraduate Medical School, Beijing 100853, People's Republic of China

* Correspondence should be addressed to C.-B.W. (email: wangcb301@126.com)

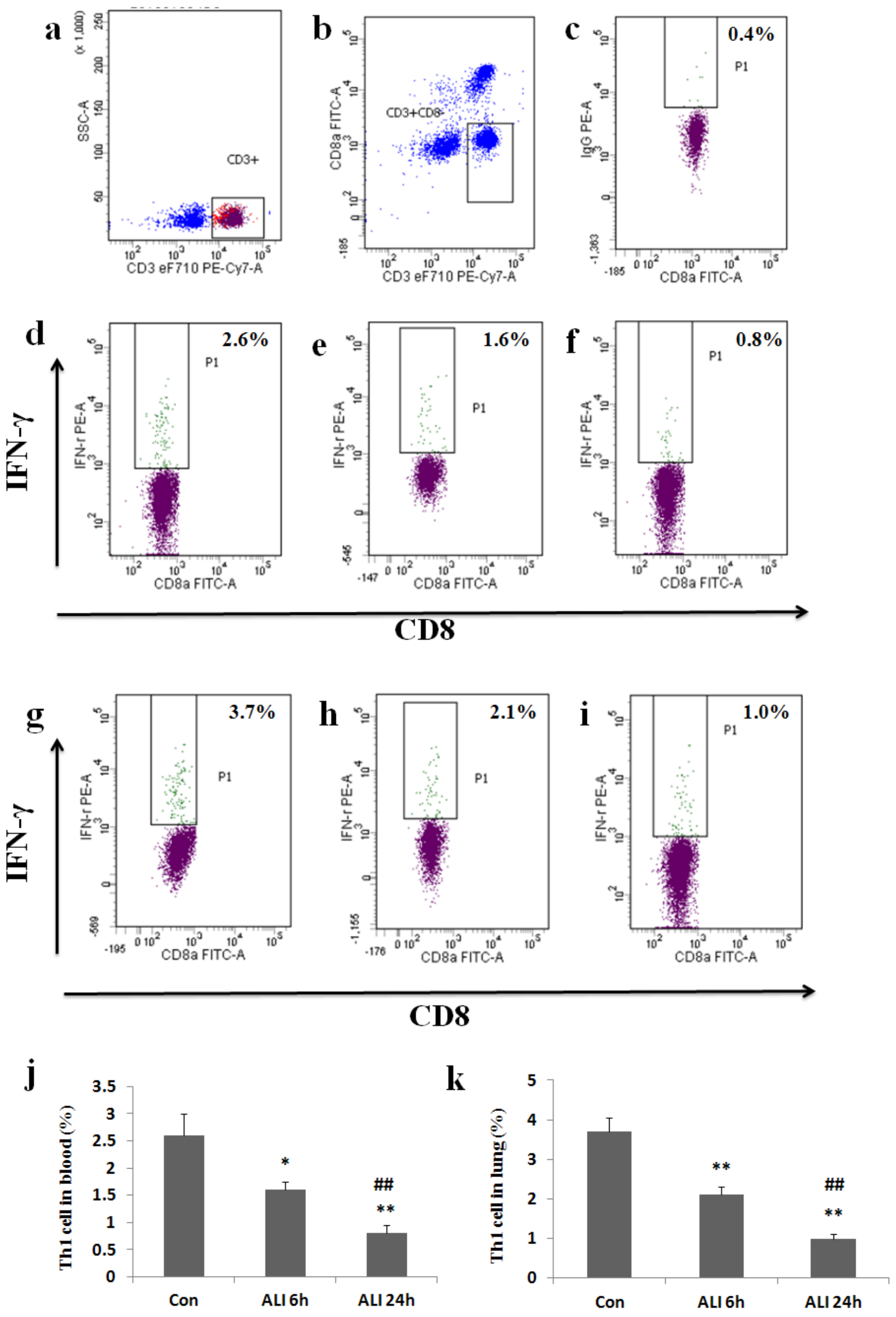

Supplementary Fig. S1. Th1 prevalence decreased in rats with smoke inhalation. Lymphocytes were stimulated with PMA, ionomycin and BFA for 5 h, and then stained with labeled antibodies as described in Materials and methods. (**a**) T lymphocytes were gated by flow cytometry; (**b**) $CD4^+$ T subsets were gated by flow cytometry; (**c**) Isotype control; (**d**) IFN-γ expression in $CD4^+$

T cells were gated by flow cytometry in Con group in peripheral blood; (**e**) IFN-γ expression in $CD4^+$ T cells were gated by flow cytometry in ALI 6h group in peripheral blood; (**f**) IFN-γ expression in $CD4^+$ T cells were gated by flow cytometry in ALI 24h group in peripheral blood; (**g**) IFN-γ expression in $CD4^+$ T cells were gated by flow cytometry in Con group in lung; (**h**) IFN-γ expression in $CD4^+$ T cells were gated by flow cytometry in ALI 6h group in lung; (**i**) IFN-γ expression in $CD4^+$ T cells were gated by flow cytometry in ALI 24h group in lung; (**j**) The circulating Th1 cell frequency was presented as mean ±S.E.M; (**k**) The Th1 cell frequency in lung was presented as mean ±S.E.M. Con (normal control group, ambient air inhalation, n=10), ALI 6h (ALI group, smoke inhalation for 6h, n=10), ALI 24h (ALI group, smoke inhalation for 24h, n=10). *P<0.05 and **P<0.01 versus normal control group; #P<0.05 and ##P<0.01 versus ALI 6h group.

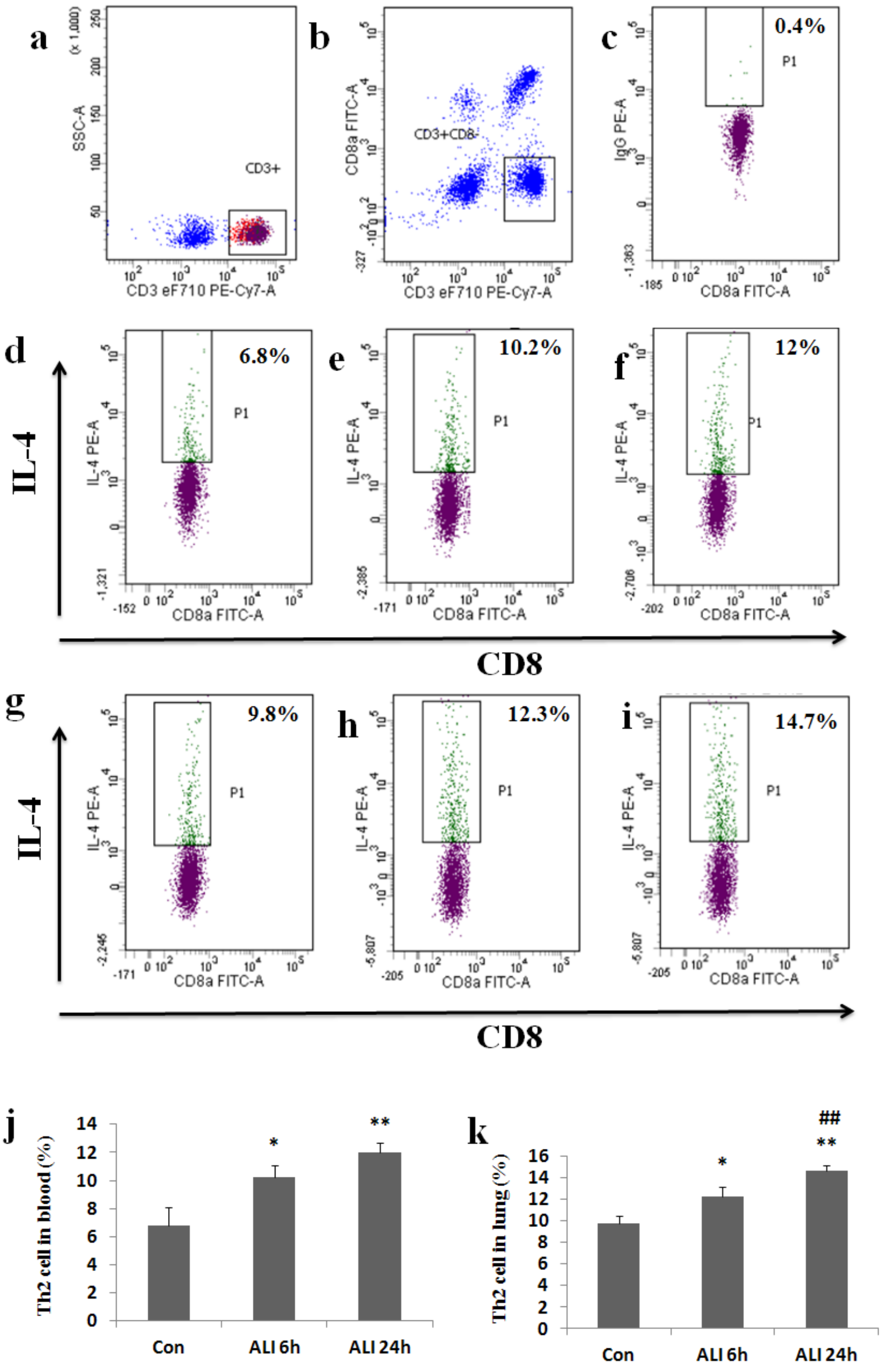

Supplementary Fig. S2. Th2 prevalence increased in rats with smoke inhalation. Lymphocytes were stimulated with PMA, ionomycin and BFA for 5 h, and then stained with labeled antibodies as described in Materials and methods. (**a**) T lymphocytes were gated by flow cytometry; (**b**) $CD4^+$ T subsets were gated by flow cytometry; (**c**) Isotype control; (**d**) IL-4 expression in $CD4^+$ T

cells were gated by flow cytometry in Con group in peripheral blood; (**e**) IL-4 expression in $CD4^+$ T cells were gated by flow cytometry in ALI 6h group in peripheral blood; (**f**) IL-4 expression in $CD4^+$ T cells were gated by flow cytometry in ALI 24h group in peripheral blood; (**g**) IL-4 expression in $CD4^+$ T cells were gated by flow cytometry in Con group in lung; (**h**) IL-4 expression in $CD4^+$ T cells were gated by flow cytometry in ALI 6h group in lung; (**i**) IL-4 expression in $CD4^+$ T cells were gated by flow cytometry in ALI 24h group in lung; (**j**) The circulating Th2 cell frequency was presented as mean ±S.E.M; (**k**) The Th2 cell frequency in lung was presented as mean ±S.E.M. Con (normal control group, ambient air inhalation, n=10), ALI 6h (ALI group, smoke inhalation for 6h, n=10), ALI 24h (ALI group, smoke inhalation for 24h, n=10). *P<0.05 and **P<0.01 versus normal control group; #P<0.05 and ##P<0.01 versus ALI 6h group.

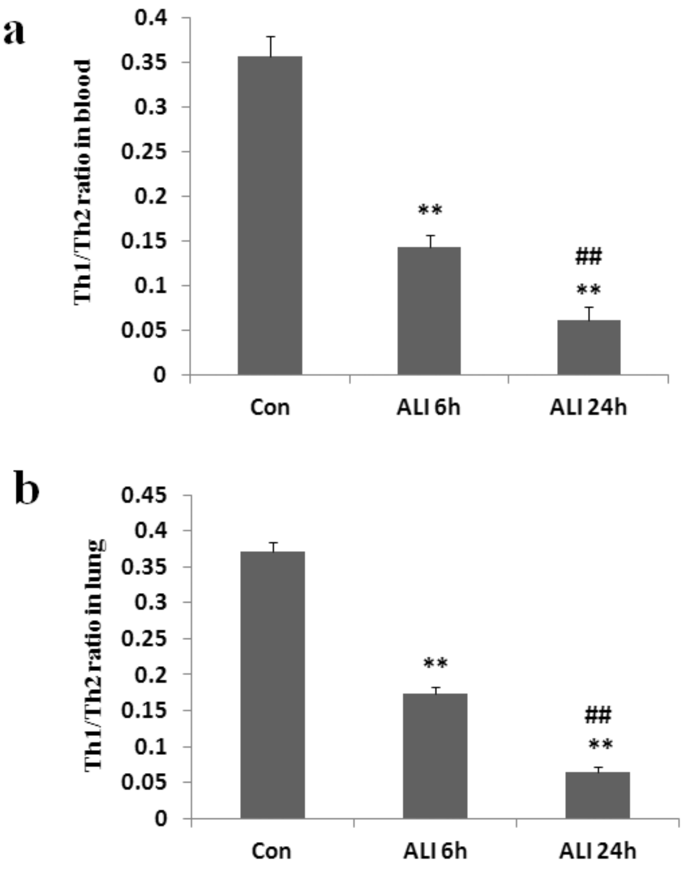

Supplementary Fig. S3. The ratio of Th1/Th2. There was a significant decrease in the ratio of Th1/Th2 in peripheral blood and lung from rats with smoke inhalation. (**a**) The ratio of Th1/Th2 in peripheral blood; (**b**) The ratio of Th1/Th2 in lung. Con (normal control group, ambient air inhalation, n=10), ALI 6h (ALI group, smoke inhalation for 6h, n=10), ALI 24h (ALI group, smoke inhalation for 24h, n=10); data are presented as mean ± S.E.M. *P<0.05 and **P<0.01 versus normal control group; #P<0.05 and ##P<0.01 versus ALI 6h group.

Supplementary Table S1. Th1- and Th2-related cytokines in serum.

Cytokines	Con	ALI 6h	ALI 24h
IFN-γ (pg/ml)	30.0±2.6	$21.0\pm4.6^{*}$	$9.2\pm1.4^{**\#}$
IL-4 (pg/ml)	6.5±1.6	$19.6\pm2.5^{**}$	$67.7\pm9.6^{**\#\#}$

Con (normal control group, ambient air inhalation, n=10), ALI 6h (ALI group, smoke inhalation for 6h, n=10), ALI 24h (ALI group, smoke inhalation for 24h, n=10); data are presented as mean ± S.E.M. *P<0.05 and **P<0.01 versus normal control group; #P<0.05 and ##P<0.01 versus ALI 6h group.

Supplementary Table S2.Th1- and Th2-related cytokines in BALF.

Cytokines	Con	ALI 6h	ALI 24h
IFN-γ (pg/ml)	35.0±4.6	19.3±3.1**	10.3±1.6**#
IL-4 (pg/ml)	6.5±1.6	19.6±2.5**	67.7±9.6**##

Con (normal control group, ambient air inhalation, n=10), ALI 6h (ALI group, smoke inhalation for 6h, n=10), ALI 24h (ALI group, smoke inhalation for 24h, n=10); data are presented as mean ± S.E.M. *P<0.05 and **P<0.01 versus normal control group; #P<0.05 and ##P<0.01 versus ALI 6h group.
